# Methods for Identifying Patients with Tropomyosin Receptor Kinase (TRK) Fusion Cancer

**DOI:** 10.1007/s12253-019-00685-2

**Published:** 2019-06-29

**Authors:** Derek Wong, Stephen Yip, Poul H. Sorensen

**Affiliations:** 1 Department of Pathology & Laboratory Medicine, University of British Columbia, Vancouver, BC Canada; 2 British Columbia Cancer Agency, Provincial Health Services Authority, Vancouver, BC Canada; 3 British Columbia Cancer Research Centre, Vancouver, BC Canada; 4 Department of Pathology, University of British Columbia, 675 West 10th Avenue, Room 4112, Vancouver, BC V5Z 1L3 Canada

**Keywords:** *NTRK* gene fusions, TRK fusions, TRK inhibitors, Next-generation sequencing, NGS

## Abstract

*NTRK* gene fusions affecting the tropomyosin receptor kinase (TRK) protein family have been found to be oncogenic drivers in a broad range of cancers. Small molecule inhibitors targeting TRK activity, such as the recently Food and Drug Administration-approved agent larotrectinib (Vitrakvi®), have shown promising efficacy and safety data in the treatment of patients with TRK fusion cancers. *NTRK* gene fusions can be detected using several different approaches, including fluorescent in situ hybridization, reverse transcription polymerase chain reaction, immunohistochemistry, next-generation sequencing, and ribonucleic acid-based multiplexed assays. Identifying patients with cancers that harbor *NTRK* gene fusions will optimize treatment outcomes by providing targeted precision therapy.

## Introduction

### TRK Receptor Family and Signaling

**Fig. 1:**
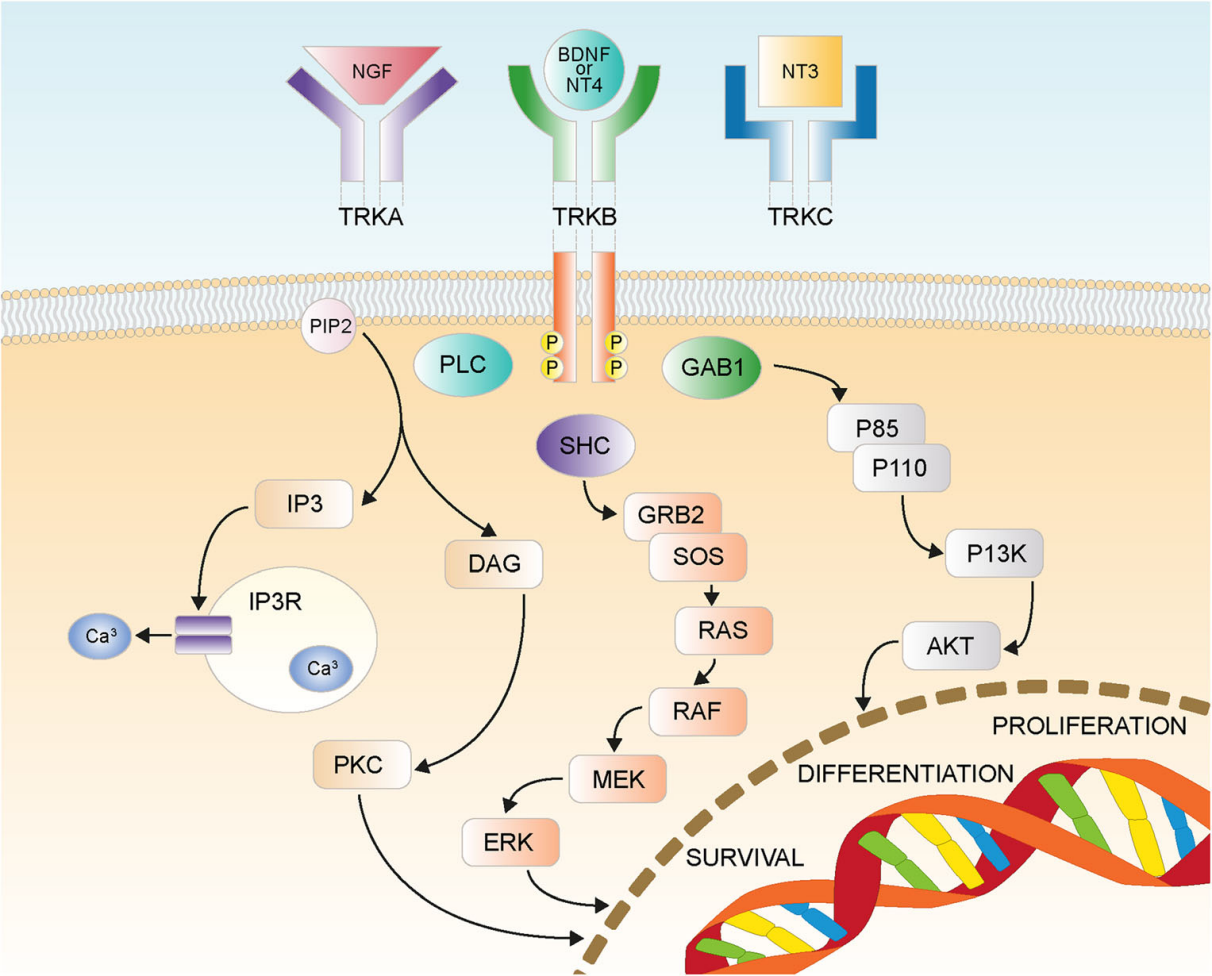
Tropomyosin receptor kinase (TRK) receptor signaling [5]. AKT, v-akt murine thymoma viral oncogene homolog; BDGF, brain-derived growth factor; DAG, diacylglycerol; ERK, extracellular signal-regulated kinase; GAB1, GRB2-associated-binding protein 1; GRB2, growth factor receptor-bound protein 2; IP3, inositol trisphosphate; MEK, mitogen-activated protein kinase; NGF, nerve growth factor; NTF-3, neurotrophin 3; PI3K, phosphatidylinositol-4,5-bisphosphate 3-kinase; PIP2, phosphatidylinositol 4,5-bisphosphate; PKC, protein kinase C; PLC, phospholipase C; RAF, rapidly accelerated fibrosarcoma kinase; RAS, rat sarcoma kinase; SHC, Src homology 2 domain containing. Reproduced with permission from Amatu A, Sartore-Bianchi A, Siena S. ESMO Open 2016;1(2):e000023

The tropomyosin receptor kinase (TRK) family is a group of three neurotrophic receptor tyrosine kinase proteins (TRKA, TRKB, and TRKC) encoded by the *NTRK1, NTRK2,* and *NTRK3* genes located on chromosomes 1q23.1, 9q21.33, and 15q25.3, respectively. These receptors are normally expressed in neuronal tissues and have high affinity for and are activated by neurotrophins. Activation of a TRK protein and subsequent signal transduction requires homo-dimerization of TRK membrane receptors following ligand binding [1]. Developmentally, TRK proteins are important for the differentiation and maturation of the central and peripheral nervous system through activation of the phosphoinositide 3-kinase/protein kinase B (PI3K-AKT) and mitogen-activated protein kinase (MAPK) signaling cascades [2–5] (Fig. 1).

### *NTRK* Gene Fusions

**Fig. 2:**
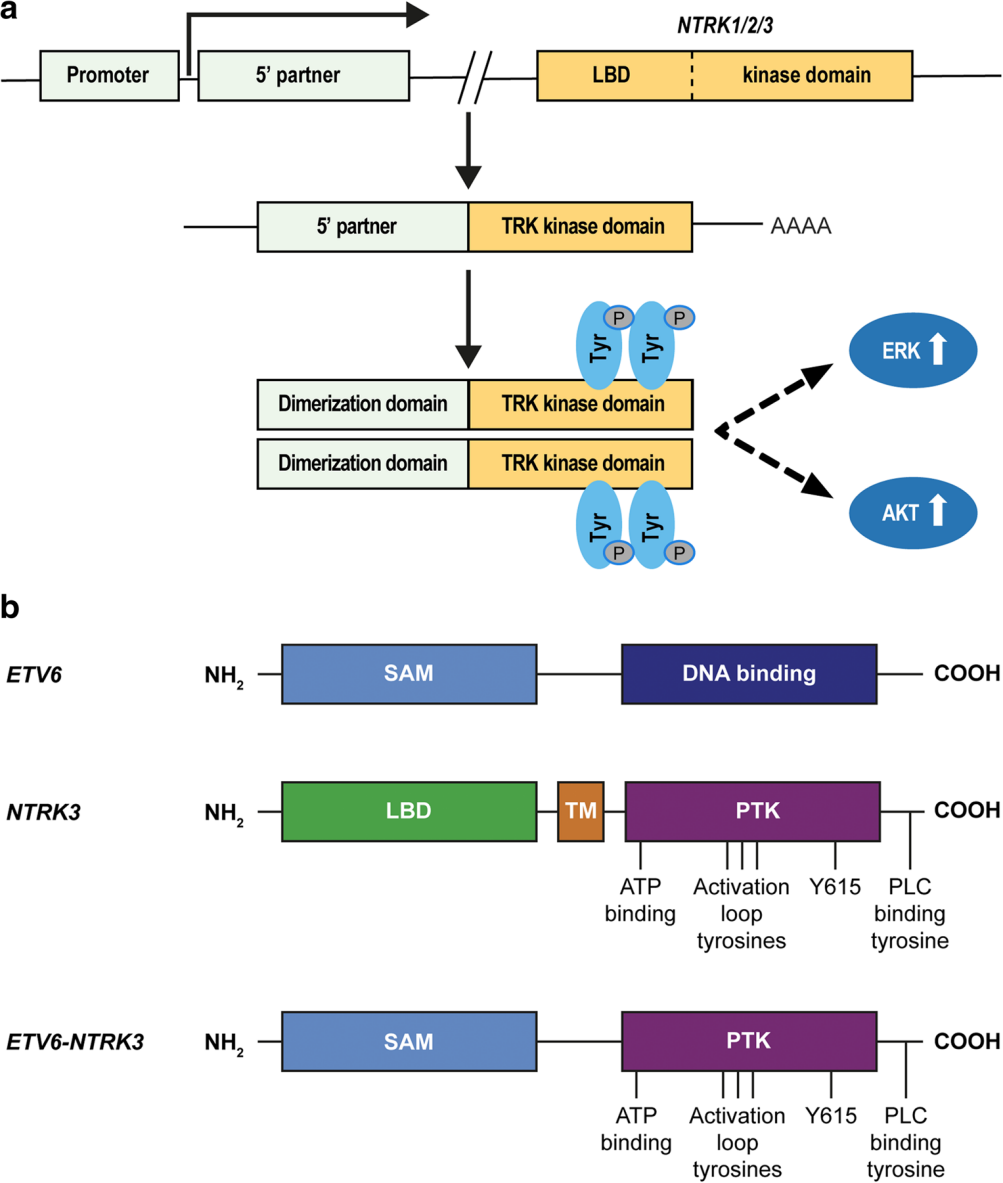
*NTRK* gene fusions. (**a**) Mechanism of *NTRK1/2/3* gene fusions; (**b**) *ETV6-NTRK3* gene fusion [9]. DNA, deoxyribonucleic acid; LBD, ligand-binding domain; PTK, tyrosine kinase; TRK, tropomyosin receptor kinase; TM, transmembrane; SAM, sterile alpha motif. Figure 2b reproduced with permission from Triche TJ, Hicks MJ, Sorensen PH. Diagnostic Pathology of Pediatric Malignancies. In: Pizzo PA, Poplack DG, editors. Principles and Practice of Pediatric Oncology, 7th Edition: Wolters Kluwer Health; 2015

Gene fusions involving the TRK protein family typically involve intra- or inter-chromosomal rearrangements of the 5′ end of a fusion partner containing a dimerization/oligomerization domain with the 3′ region of an *NTRK* gene encoding the tyrosine kinase domain. The resulting fusion gene leads to the expression of a chimeric protein that lacks the TRK ligand-binding domain but retains the tyrosine kinase domain. This fusion protein harbors oncogenic and transforming potential through overexpression and constitutive activation of the TRK kinase domain due to the presence of a dimerization domain derived from the fusion partner [5–8] (Fig. 2a). Historically, the first *NTRK* gene fusion was isolated from a human colon carcinoma by classical deoxyribonucleic acid (DNA) transformation assays [10]. The *ETV6-NTRK3* gene fusion is the most extensively studied *NTRK* gene fusion. Recurrent *NTRK* gene fusions involving *ETV6* and *NTRK3* (Fig. 2b) were first identified in infantile (or congenital) fibrosarcoma, a malignant tumor of fibroblasts that occur in patients aged 2 years or younger [11], and then shortly after in congenital mesoblastic nephroma, the renal counterpart of infantile fibrosarcoma [12, 13]. Since then, *ETV6-NTRK3* fusions have been identified in numerous other cancer types, including secretory breast carcinoma [14], acute myeloid leukemia [15], radiation-associated thyroid cancer [16], pediatric high-grade glioma [17], Philadelphia chromosome-like ALL, and other tumor types (Table 1).

**Table 1:** Summary of *NTRK* gene fusions by detection method and tumor type

Detection/validation method(s)^a^	Tumor type (*NTRK* gene fusion)	Study
FISH	Acute myeloid leukemia (*ETV6-NTRK3*)	Eguchi et al. [15]
	Congenital mesoblastic nephroma (*ETV6-NTRK3*)	El Demellawy et al. [18]
RT-PCR	Congenital mesoblastic nephroma (*ETV6-NTRK3*)	Knezevich et al. [12]
	Infantile fibrosarcoma (*ETV6-NTRK3*)	
RT-PCR and FISH	Congenital mesoblastic nephroma (*ETV6-NTRK3*)	Rubin et al. [13]
	MASC (*ETV6-NTRK3*)	Skalova et al. [19]
RT-PCR and IHC	Infantile fibrosarcoma (*ETV6-NTRK3*)	Bourgeois et al. [20]
IHC and FISH	Colorectal cancer (*LMNA-NTRK1*)	Sartore-Bianchi et al. [21]
NGS (DNA- or RNA-seq), FISH, or IHC	MASC (*ETV6-NTRK3*)	Drilon et al. [22]
	Colorectal cancer (*LMNA-NTRK1*)	
	Glioneuronal tumor (*BCAN-NTRK1*)	
	Lung cancer (*SQSTM1-NTRK1*)	
NGS^b^ or FISH	Lung cancer (*IRF2BP2-NTRK1*)	Drilon et al. [23];
	Melanoma (*GON4L-NTRK1, TRIM63-NTRK1*)	
	Pancreatic cancer (*CTRC-NTRK1*)	
	Thyroid cancer (*IRF2BP2-NTRK1, PPL-NTRK1, TPM3-NTRK1*)	
	Soft tissue sarcoma (*TPM3-NTRK1, TPM4-NTRK3*)	Kummar and Lassen [24]
	Appendiceal cancer (*LMNA-NTRK1*)	
	Breast cancer (*GATAD2B-NTRK1, LMNA-NTRK1, TPM3-NTRK1*)	
	Cholangiocarcinoma (*LMNA-NTRK1, TPM3-NTRK1*)	
	Colon cancer (*PLEKHA6-NTRK1*)	
	Infantile fibrosarcoma (*SQSTM1-NTRK1, TPM3-NTRK1*)	
NGS (DNA-seq) and IHC	Melanoma (*GON4L-NTRK1, TRIM63-NTRK1, TRAF2-NTRK2, DDR2-NTRK1)*	Lezcano et al. [25]
NGS (RNA-seq) and array-comparative genome hybridization	Ganglioglioma (*TLE4-NTRK2*)	Prabhakaran et al. [26]
NGS (DNA-seq and/or RNA-seq)	Fibrous tumor (*TFG-NTRK3*)	Chmielecki et al. [27]
	Infantile fibrosarcoma (*SQSTM1-NTRK1*)	
NGS (DNA- or RNA-seq) and IHC	Lung cancer (*IRF2BP2-NTRK1, MRPL24-NTRK1, P2RY8- NTRK1*)	Hechtman et al. [28]
	Melanoma (*TRIM63-NTRK1, TRAF2-NTRK2*)	
	Soft tissue sarcoma (*TPM4-NTRK3*)	
	Colorectal cancer (*LMNA-NTRK1, ETV6-NTRK3*)	
	Glioblastoma (*BCR-NTRK2, ZNF710-NTRK3*)	
NGS (RNA-seq), IHC, and FISH	Colorectal cancer (*SCYL3-NTRK1*)	Milione et al. [29]
NGS (RNA-seq)	Pancreatic cancer (*CEL-NTRK1*)	Edgren et al. [30]
	Glioblastoma (*BCAN-NTRK1, NFASC-NTRK1*)	Kim et al. [31]
	Neuroendocrine cancer (*ETV6-NTRK3*)	Sigal et al. [32]
	Lung cancer (*IRF2BP2-NTRK1, TRIM24-NTRK2*)	Stransky et al. [33]
	Thyroid cancer (*IRF2BP2-NTRK1, TFG-NTRK1, RBPMS-NTRK3*)	
	Squamous cell cancer of the head and neck (*PAN3-NTRK2, ETV6-NTRK3*)	
	Sarcoma (*TPM3-NTRK1*)	
	Glioma (*AFAP1-NTRK2, SQSTM1-NTRK2*)	
	Glioblastoma (*NFASC-NTRK1*)	
NGS (RNA-seq) and FISH	Congenital mesoblastic nephroma (*EML4-NTRK3*)	Church et al. [34]
	Infantile fibrosarcoma (*EML4-NTRK3*)	
NGS (RNA-seq), IHC, and FISH	Colorectal cancer (*TPM3-NTRK1*)	Lee et al. [35]
NGS (RNA-seq), FISH, and RT-PCR	GIST (*ETV6-NTRK3*)	Brenca et al. [36]
NGS (whole-genome sequencing and RNA-seq)	Glioma (*AKAP13-NTRK3*)	Yoshihara et al. [37]
	Astrocytoma (*NACC2-NTRK2, QK1-NTRK2*)	Jones et al. [38]
	Glioma (*TPM3-NTRK1, AGBL4-NTRK2, VCL-NTRK2, BTBD1- NTRK3*)	Wu et al. [17]
NGS (whole-exome sequencing, whole-genome sequencing, and/or RNA-seq)	Acute lymphoblastic leukemia (*ETV6-NTRK3*)	Roberts et al. [39]
	Large cell neuroendocrine cancer (*COP1-NTRK1*)	George et al. [40]
NGS (whole-genome sequencing and RNA-seq)	Thyroid cancer (*TPM3-NTRK1*)	Ronsley et al. [41]
Targeted NGS (DNA-seq)	Uterine endometrial cancer (*LRRC71-NTRK1*)	Hartmaier et al. [42]
	Lung cancer (*GRIPAP1-NTRK1*)	
	Intrahepatic cholangiocarcinoma (*RABGAP1L-NTRK1*)	Ross et al. [43]
	GIST (*ETV6-NTRK3*)	Shi et al. [44]
	Spitzoid neoplasm (*TP53-NTRK1, LMNA-NTRK1*)	Wiesner et al. [45]
	Lung cancer (*TPM3-NTRK1*)	Zheng et al. [46]
	Thyroid cancer (*PPL-NTRK1*) Glioblastoma (*ARHGEF2-NTRK1, CHTOP-NTRK1*)	
Targeted NGS (DNA- and RNA-seq)	Thyroid cancer (*EML4-NTRK3, SQSTM1-NTRK3, IRF2BP2- NTRK1*)	Liang et al. [47]
Targeted NGS (DNA-seq) or FISH	Lung cancer (*CD74-NTRK1, MPRIP-NTRK1*)	Vaishnavi et al. [48]
Targeted NGS (DNA- or RNA-seq) and/or FISH	Uterine sarcoma (*LMNA-NTRK1*, *TPM3-NTRK1*, *RBPMS-NTRK3*)	Chiang et al. [49]

^a^Detection/validation method(s) used in study to identify *NTRK* gene fusion(s)

^b^Specific NGS method used in study not specified. DNA, deoxyribonucleic acid; FISH, fluorescent in situ hybridization; IHC, immunohistochemistry; MASC, mammary analog secretory carcinoma; NGS, next-generation sequencing; RNA, ribonucleic acid; RT-PCR, reverse transcription polymerase chain reaction

Studies investigating the mechanism of *ETV6-NTRK3* transformation in NIH 3T3 cells and other fibroblasts have revealed that autophosphorylation of this chimeric protein results in the dual activation of RAS-ERK1/2 and PI3K-AKT signaling, and is dependent on homo- and hetero-dimerization mediated by the dimerization domain of *ETV6* [7, 50, 51]. Expression of the *ETV6-NTRK3* fusion in mammary tissues of mice has also identified early breast progenitor cells rather than stem cells as the direct targets of transformation and has provided valuable models for preclinical studies [52]. Interestingly, the protein encoded by the *ETV6-NTRK3* fusion has also been found to interact with and be dependent on the activity of insulin-like growth factor 1 receptor (IGF1R) for both stability and transformation, which may provide another clinical avenue for future treatments [53–55].

Although the *ETV6-NTRK3* fusion is the most extensively studied *NTRK* gene fusion, fusion events involving all three of the *NTRK* genes and over 50 different 5′ fusion partners have been identified (Table 1). *NTRK* gene fusions have been found in over 20 different cancer types and in up to 1% of all solid tumor malignancies, suggesting that *NTRK* gene fusions may be oncogenic drivers regardless of the tumor type [8].

### Clinical Data for TRK Inhibitors

Fusions involving TRK proteins lead to constitutive activation of the kinase domain similarly to many other oncogenic drivers such as *BCR-ABL* translocation and *EGFR* amplification/mutation. One strategy in targeting these kinds of oncogenic drivers has been to develop small molecule inhibitors to block the downstream signaling pathways that are activated and drive the cancer. Currently, there are several small molecular inhibitors targeting TRK in phase II clinical trials; the most notable are larotrectinib, a highly selective TRK inhibitor (TRKA/B/C), and entrectinib, a broader tyrosine kinase inhibitor (*TRKA/B/C, ROS1, ALK*) [5, 56]. Both have demonstrated the ability to cross the blood-brain barrier, making them suitable agents for central nervous system (CNS) tumors that harbor TRK fusions [57, 58]. Clinical basket trials using larotrectinib (NCT02122913, NCT02637687, NCT02576431) have shown durable overall response rates of 93% in a pediatric phase I/II trial and 75% in a combined adult and pediatric phase I/II trial [23, 59]. Adverse events were predominantly grade 1 or grade 2 with no grade 3 or grade 4 adverse events attributable to larotrectinib seen in more than 5% of the patients regardless of tumor type or fusion partner [23, 59]. These data clearly demonstrate the potency of larotrectinib as a therapeutic option for patients that harbor *NTRK* gene fusions. Similarly, phase I/IIa clinical trials for entrectinib demonstrated low toxicity with reversal of side-effects following dose monitoring [22]. Larotrectinib has been recently approved for use in the United States for patients with solid tumors that harbor an *NTRK* fusion gene [60]. Clinical trials for both drugs are still currently on-going and further support the utility of identifying TRK fusion cancers in order to provide effective and durable clinical therapeutic options to patients. In this review, we summarize the methods available for detecting *NTRK* gene fusions in cancer.

### Methods to Identify Patients with TRK Fusion Cancer

Several different approaches have been used to detect the presence of *NTRK* gene fusions at the DNA and ribonucleic acid (RNA) level and TRK expression. These methods include traditional clinical assays such as fluorescent in situ hybridization (FISH), reverse transcription polymerase chain reaction (RT-PCR), immunohistochemistry (IHC), and newer, emerging technologies such as next-generation sequencing (NGS) and RNA-based multiplexed assays (Nanostring). Each technique is associated with advantages and disadvantages (Table 2).

**Table 2:** Advantages and disadvantages of methodologies for detecting tropomyosin receptor kinase (TRK) fusion cancer

	Fluorescence in situ hybridization (FISH)	Reverse transcription polymerase chain reaction (RT-PCR)	Pan-TRK immunohistochemistry (IHC)	Next-generation sequencing (NGS)
Advantages	• Location of the target within the cell can be detected [61]	• High sensitivity and specificity [20, 62]	• Inexpensive [63, 64]	• Detection of novel fusion partners [34] and fusions expressed at RNA level [65]
			• Decentralized, available in most laboratories [20]	
	• High sensitivity and specificity [63]	• Assays detect fusions expressed at the RNA level [62]	• Established reimbursement codes [66]	• Ability to test multiple actionable targets simultaneously [34]
	• Several fluorophores can be used at once to detect different targets in one sample [67]	• Inexpensive [68]	• Turnaround time: ~2 days [64]	• Plays key role in diagnostic work-up of TRK fusion cancer [23]
				• Relevance of NGS increases as number of actionable targets grows [69]
				• High sensitivity and specificity potential [63]
Disadvantages	• Requires fluorescence microscopy [61]	• Target sequences must be known; unable to detect novel fusion partners [34, 68]	• Cannot differentiate between fusion and wild-type TRK expression [28]	• Turnaround time: ~1–3 weeks [69]
				• Technically complex and costly [70]
	• Target sequence must be known; unable to detect novel fusion partners unless break-apart probes are used [34]	• Development of separate tests required for each *NTRK* gene [71]	• Scoring algorithms are not standardized [20]	• Requires highly centralized testing model [34] and bioinformatics infrastructure [72]
			• Additional testing required to determine course of action [28]	• Reimbursement currently restricted [73]
	• Development of separate tests required for each *NTRK* gene [71]			
	• Cannot demonstrate that functional protein has been generated [34, 67]			
				• Sensitivity and specificity of NGS assays vary widely [63, 74]

### FISH

**Fig. 3:**
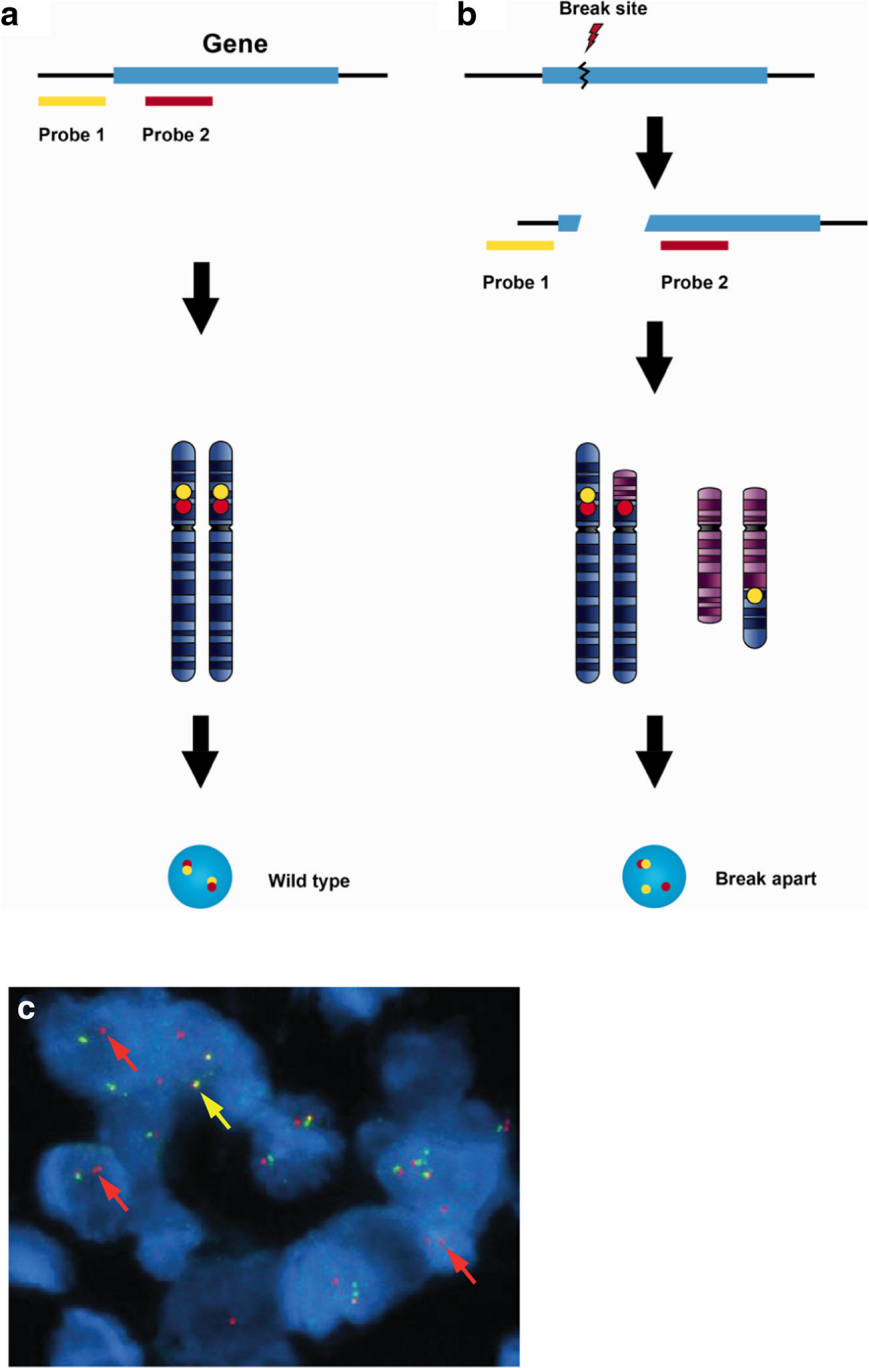
Break-apart fluorescent in situ hybridization (FISH). (**a**) The wildtype pattern shows two pairs of closely situated or fused signals. (**b**) In break-apart FISH, a set of probes specific for the target gene is used. When translocation occurs involving a breakpoint between the two probe sites, the loci split apart. (**c**) An example of break-apart FISH testing results in a patient with soft-tissue sarcoma and an *LMNA-NTRK1* gene fusion [76]. *NTRK1* break-apart FISH demonstrates both paired green (5′ *NTRK1*) and red (3′ *NTRK1*) signals corresponding to the normal *NTRK1* gene (yellow arrow). Isolated red signals (red arrows) are observed in tumor nuclei (stained blue with DAPI) indicative of a chromosomal deletion leading to an *NTRK1* gene fusion. DAPI, 4′,6-diamidino-2-phenylindole. Figures 3a and b reproduced with permission from Cheng L, Zhang S, Wang L, MacLennan GT, Davidson DD. J Pathol Clin Res 2017;3(2):73–99. Figure 3c reproduced with permission from Doebele RC, Davis LE, Vaishnavi A, Le AT, Estrada-Bernal A, Keysar S, et al. Cancer Discov 2015;5(10):1049–57

Historically, FISH has been the gold standard for the clinical detection of gene fusions (e.g. *BCR-ABL* rearrangements in chronic myeloid leukemia) [75]. FISH uses fluorescently labeled RNA or DNA probes that bind to complementary sequences on formalin-fixed paraffin embedded (FFPE) tumor samples. For gene fusions typical of certain malignancies (e.g. *ETV6-NTRK3* in infantile fibrosarcoma), dual color FISH probes can be used. One major advantage of FISH analysis is the ability to detect the presence of a fusion event involving a target gene without prior knowledge of the fusion partner by utilizing “break-apart” probes, where each probe is directed to the 5′ and 3′ ends of the target gene, respectively (Fig. 3a and b). An intact *NTRK* gene would result in overlapping probes and produce yellow fluorescence whereas a translocation event would result in the probes “breaking apart” to produce two individual probes (red and green) indicating a break in the gene most likely arising from a chromosomal translocation. An example is the detection of a *LMNA-NTRK1* fusion using break-apart probes in a soft-tissue sarcoma [76] (Fig. 3c). Although this method is useful when the fusion partner is unknown, individual FISH analysis must be performed for each of the three *NTRK* genes due to the sequence specificity of the probes; this can be labor- and cost-intensive. Lastly, due to the detection of gene rearrangements at the DNA level, FISH does not provide any information as to whether an oncogenic fusion protein is produced.

### RT-PCR

**Fig. 4:**
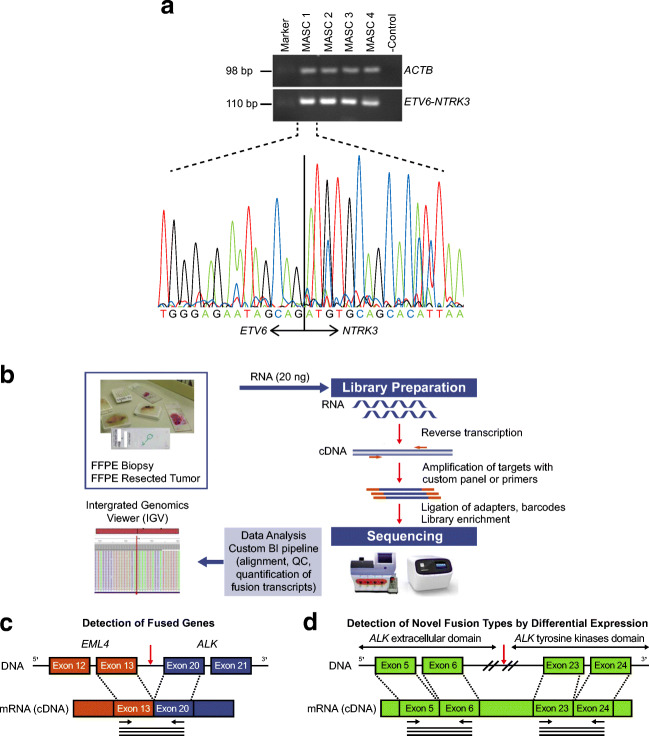
Reverse transcription polymerase chain reaction (RT-PCR) and next-generation sequencing (NGS). (**a**) Example results for reverse transcription polymerase chain reaction (RT-PCR) testing [80]. RT-PCR for *ETV6-NTRK3* fusion transcripts in mammary analogue secretory carcinoma (MASC) tumors. ACTB, ß-actin, MASC 1, MASC 2, MASC 3 and MASC 4, tumor samples from Case 1, Case 2, Case 3, and Case 4, respectively. (**b**–**d**) Summary of NGS [79]. *(B) RNA* is extracted from formalin-fixed, paraffin-embedded (FFPE) tumor specimens and reverse transcribed into complementary DNA (cDNA). The cDNA is amplified with a panel of primers targeting fusion and native control transcripts. The resulting libraries are sequenced on Ion Torrent instruments and the sequence reads are then enumerated using a custom pipeline. Identified fusion transcripts are confirmed in the Integrative Genomics Viewer (IGV) to check that sequence reads span both fusion partners. (C) Fused genes are detected by PCR amplicons that span a known fusion breakpoint. (D) Novel fusions may also be detected based on overexpression of the kinase domain of selected targets. Figure 4a reproduced with permission from Fehr A, Loning T, Stenman G. Am J Surg Pathol 2011;35(10):1600–2. URL: https://journals.lww.com/ajsp/Citation/2011/10000/Mammary_Analogue_Secretory_Carcinoma_of_the.20.aspx. Figures 4**b**–**d** reproduced with permission from Beadling C, et al. J Mol Diagnostics. 2016;18(2):165–175

Tumor RNA from fresh frozen or FFPE samples can be extracted and converted to complementary DNA (cDNA) sequences using reverse transcription. The cDNA is then amplified using polymerase chain reaction (PCR) primers that are located on either side of the fusion breakpoint, resulting in a PCR amplification product only when that specific fusion is present. The amplification products can then be visualized using intercalating dyes that bind to double-stranded DNA or a fluorescent reporter-quencher system that allows for multiplexing of multiple primer sets [77]. RT-PCR provides a highly specific, rapid, economical, and sensitive testing method, even at low transcript levels, with quick turnaround time and multiplexing capabilities [78] compared with FISH analysis. However, RT-PCR requires prior knowledge of the fusion partners. A variation of RT-PCR that can detect the presence of a fusion with an unknown partner has been developed [79]. Although RT-PCR has a quick turnaround time once established, the design and validation of each primer set is labor intensive, more so when multiplexing multiple primer sets, which introduces the potential for cross-interactions. Robust detection by RT-PCR also relies on the quality of the RNA extracted, which can vary greatly due to the unstable nature of RNA. An example of the detection of an *ETV6-NTRK3* fusion in a mammary analog secretory carcinoma using RT-PCR is presented in Fig. 4a [80].

### IHC

**Fig. 5:**
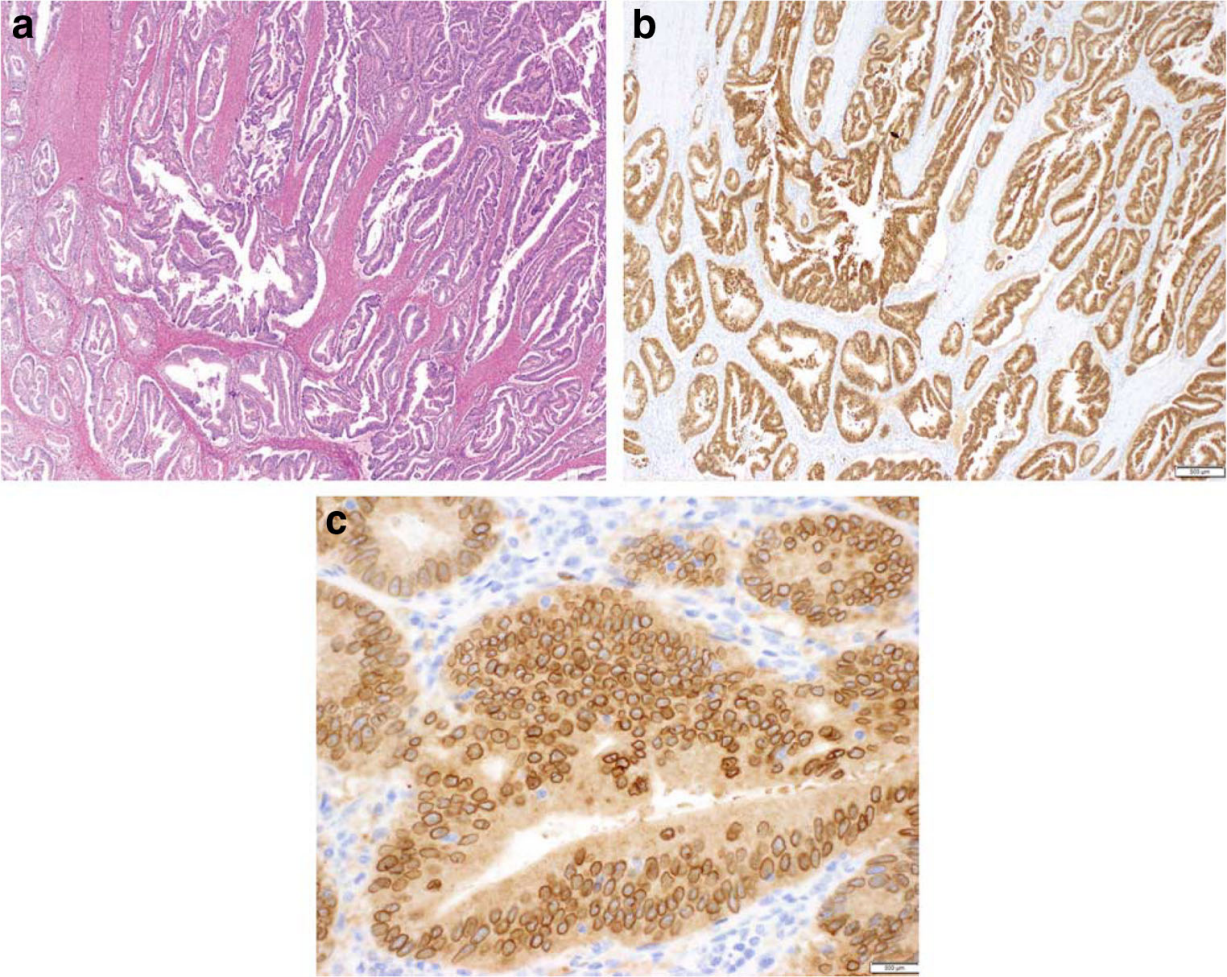
Example pan-tropomyosin receptor kinase (TRK) immunohistochemistry (IHC) staining pattern in a patient with colorectal carcinoma with an ***LMNA-NTRK1*** fusion [28]. A moderately differentiated colorectal carcinoma with conventional histology (hematoxylin and eosin) and an *LMNA* exon 12-*NTRK1* exon 12 fusion (A) displays diffuse cytoplasmic and nuclear membrane staining for pan-TRK IHC (pan-TRK IHC clone EPR17341, Abcam, Cambridge, MA) (B, C). Reproduced with permission from Hechtman JF, Benayed R, Hyman DM, Drilon A, Zehir A, Frosina D, et al. Am J Surg Pathol 2017;41(11):1547–51. URL: https://journals.lww.com/ajsp/Abstract/2017/11000/Pan_Trk_Immunohistochemistry_Is_an_Efficient_and.13.aspx

While FISH and RT-PCR are used to detect fusions at the DNA and RNA level, respectively, IHC can be used to survey the protein expression of your target of interest using antibodies tagged with a colorimetric label. In contrast to FISH, the availability of a pan-TRK antibody eliminates the need to perform individual assays for each TRK protein. The use of a pan-TRK monoclonal antibody has been shown to be sensitive and reliable, identifying TRK expression in 20/21 cases in one study [28] and 21/28 cases in another study [81]. In the second study, pan-TRK IHC was less effective in detecting fusions involving *NTRK3*, which may reduce the overall sensitivity of IHC as the *ETV6-NTRK3* fusions has been reported as the most common TRK fusion in pan-cancer studies [33, 81]. An example of the detection of a protein resulting from the *LMNA-NTRK1* fusion using IHC can be found in Fig. 5 [28]. A pan-TRK monoclonal antibody has been recently approved for in vitro diagnostic use (Ventana Medical Systems), which should provide a more reproducible reagent for the detection of TRK expression. One caveat when using IHC to detect TRK is that the antibody does not discriminate between expression of the wildtype and fusion protein. Therefore, strong staining may indicate either expression of the wildtype protein or the presence of a TRK fusion protein. Interpretation of IHC results can also be subjective due to the heterogeneity of normal tissue expression and thus requires strict controls. However, IHC remains a cost- and sample-effective method with quick turnaround times. Moreover, IHC is commonly used and widely available in most pathology laboratories and may be an effective initial screening step for TRK fusions prior to confirmation with a secondary method such as NGS.

### NGS and Other Multiplexed Assays

The most comprehensive and inclusive method of identifying fusions is through NGS assays such as whole genome, targeted panel, and RNA sequencing (Fig. 4b–d). As the power of NGS technology has increased, the cost of analyzing each sample has also decreased. However, with all NGS-based assays, the need for analytic and bioinformatic support may be prohibitive for many laboratories. Although not primarily used for the detection of fusions, whole genome studies have led to the discovery of novel, recurrent, and rare fusions [82]. The usefulness of these data is limited because, on the genomic level, fusions are often found as passengers of general genomic instability, a hallmark of cancer. However, whole genome, in conjunction with RNA sequencing, has been integral for validating and determining the biological relevance and potential downstream effects of genomic fusions [82, 83]. Whole-genome sequencing has clear benefits and uses in terms of fusion discovery and basic biology research, although they are currently not suitable as a universal method in a clinical setting due to the intensive bioinformatics required to interpret the data generated.

The most common method of detecting fusion events utilizing NGS is through analyzing a specific panel of genes. The genes to be sequenced are isolated by either an amplicon-based or hybrid capture methodology. Amplicon-based methods enrich for target genes by PCR amplification of a distinct set of genes which requires less input DNA but can only detect fusion partners that are also included in the panel. In contrast, hybrid capture enrichment targets specific genomic regions through hybridization with a substrate (streptavidin/biotin), and thus can be used to identify an unknown fusion linked to the target sequence. Detection can still be challenging with low complexity sequencing, as the breakpoint may be located within a large intron, with adequate coverage costly and potentially unfeasible. This is particularly problematic with *NTRK2* and *NTRK3* fusion testing by DNA sequencing, with the entire intron sequence needing to be included in testing panels. Lastly, RNA-based panel sequencing provides the most utility by enriching for specific expressed transcripts without the complication of large introns. These panels, such as the Illumina TruSight 170 panel (TST170) and the Archer FusionPlex assay, are designed to target and enrich for hundreds of fusions involving specific genes using hybrid capture and anchored multiplex PCR technologies, respectively [46]. The advantage of this technology is that knowledge of only one of the partners is required, allowing for the potential discovery of novel fusion partners. The use of messenger RNA (mRNA) also provides confidence that the fusion is expressed. However, this method is still limited in that one of the partner genes must be present on the panel.

Although the turnaround time for NGS technology can be long (6–21 days), it provides an extremely comprehensive, specific, and sensitive technique with extensive multiplex capabilities. Many of the larger clinical laboratories have moved towards integrating NGS testing into routine clinical workups such as the Memorial Sloan Kettering-Integrated Mutation Profiling of Actionable Cancer Targets (MSK-IMPACT), which has been used since 2014 and was approved by the Food and Drug Administration (FDA) in 2017. The MSK-IMPACT panel is hybrid-capture-based and includes 341 key cancer genes of which 14 are recurrently rearranged genes [69]. However, for many clinical laboratories, the bioinformatics demands, costs, and availability of NGS facilities/personnel can be prohibitive.

Lastly, although not considered NGS, the Nanostring nCounter Vantage 3D is an extensively multiplexed high-throughput hybridization assay that uses target-specific probes to detect fusion transcripts. Although the Nanostring platform requires more input RNA (~100–300 ng) compared to NGS-based panels (~10–100 ng), this technique does not introduce PCR amplification biases or sequencing errors since the assay uses native, unamplified RNA. Instead, the Nanostring platform detects fusions by using probes that are designed to bind directly to the fusion junction. Therefore, fusions that occur at non-canonical breakpoints or are not present in the panel would not be detected. At this point in time, the existing fusion platform only tests for two specific *NTRK1* fusions, although additional assays can be created to detect the full complement of known *NTRK* gene fusions.

### Summary of Testing Methods Used to Identify Patients with TRK Fusion Cancer

Due to the large variability in *NTRK* gene fusion partners and the limitations of many of the available testing methods, testing for *NTRK* gene fusions often makes use of two independent testing methods in order to provide a reliable diagnosis [81]. The method used by nearly all studies has been RNA-based NGS due to the comprehensive and extensively multiplexed nature of this technology. Several studies including the phase I trials for entrectinib have used RNA-based anchor multiplexed NGS to identify gene fusion events in patients followed by FISH confirmation with break-apart probes [84] or IHC [85]. Other studies including the phase II basket STARTRK-2 trial have used a two-step IHC-NGS technique [86]. In this method, IHC screening is performed using a cocktail of pan-tyrosine kinase antibodies that detect expression of TRKA, TRKB, TRKC, ROS1, and ALK followed by NGS using RNA-based anchored multiplex PCR to determine the exact fusion, similarly to the previously mentioned studies. This two-step diagnostic test using IHC as an initial screen followed by NGS appears to be a quick and cost-effective method for screening out *NTRK* fusion-negative cases. A caveat is that strong IHC staining may also indicate overexpression of wildtype TRK proteins, requiring validation by NGS [86]. Indeed, strong wildtype TRK protein detection is a major caveat of using antibodies alone, which do not discern between wildtype proteins and TRK fusion proteins. Issues with the scoring methods used by pathologists may also lead to these discrepancies with IHC alone. However, if the goal is to solely detect *NTRK* fusions leading to enhanced TRK expression, the availability of a reliable pan-TRK antibody would be a very useful first step for increasing the detection rate.

### *NTRK* Fusion Testing in Clinical Practice: Challenges and Future Perspectives

Data from the larotrectinib and entrectinib phase I and II clinical trials have shown durable benefit and well-tolerated toxicity, which warrants the introduction of routine testing for *NTRK* gene fusions. However, challenges persist in incorporating these tests into routine laboratory diagnostics. Notably, oncogenic *NTRK* gene fusions have been identified in ~1% of solid tumors [81]. Therefore, routine testing may be limited to tumors where canonical driver mutations are not identified. However, in cancers that are typically driven by oncogenic fusions, including sarcomas, use of targeted sequencing platforms such as Childseq [87], or more recently the extensively multiplexed Nanostring platform, has already been shown to be more cost-effective and comparably reliable in identifying the driver fusion compared to more traditional methods such as IHC, FISH, and RT-PCR [88]. Recent NGS panels such as the Illumina Trusight fusion panel [89] and MSK-IMPACT [90] have also been found to be as effective in detecting gene fusion events involving *ROS1, ALK,* and *RET* fusions. These studies provide support towards using NGS molecular assays as routine diagnostic tests in preference to more traditional methods as the costs become lower and the testing panels become more inclusive.

Traditional methods often suffer from limitations. IHC relies on the availability and efficacy of antibodies, which can vary greatly from lot-to-lot and does not conclusively identify the presence of a fusion protein. However, this variability might be mitigated by the availability of the pan-TRK IHC assay from Ventana. FISH relies on human interpretation of the fluorescent signals and cannot be multiplexed, and RT-PCR requires knowledge of the fusion junction. The greatest barriers to routine use of NGS assays are the need for stringent validation of results over the traditional methods and the bioinformatic pipeline/expertise required for analyzing the data. Many companies and third parties have begun to address the bioinformatic issue by creating user-friendly software to accompany their assays, allowing for rapid and straightforward analysis of the data generated. Although globally available, country and regional variations in access to NGS testing and the high costs of testing may pose challenges for ensuring broad patient access to these tests.

## Conclusions

*NTRK* gene fusions have garnered much clinical attention recent years due to the efficacy of small molecule inhibitors such as the recently FDA-approved use of larotrectinib (Vitrakvi®) and entrectinib in clinical trials. These drugs have also shown penetrance through the blood-brain barrier which will provide much-needed therapeutic options to patients with CNS malignancies, a field which has struggled to find durable and effective treatment options. *NTRK* gene fusions have been identified using several different approaches including FISH, RT-PCR, and NGS. IHC provides a useful screening technique to identify tumors with potential *NTRK* gene fusions that warrant further confirmation with NGS or other robust techniques, but there is a need to overcome the lack of sensitivity to detect fusions involving *NTRK3*. *NTRK* gene fusions have been identified in a broad range of cancers and appear to be tumor agnostic driver events. Although they may only be present in a small proportion of tumors, identifying these patients will be crucial for providing precision therapeutic options going forward. Therefore, robust testing methods are essential to identify the patients that harbor TRK fusion cancer in order to provide them with the benefit of precision medicine.

## References

[1] Maruyama I (2014). Mechanisms of activation of receptor tyrosine kinases: monomers or dimers. Cells.

[2] Arevalo JC, Wu SH (2006). Neurotrophin signaling: many exciting surprises!. Cell Mol Life Sci.

[3] Nakagawara A (2001). Trk receptor tyrosine kinases: a bridge between cancer and neural development. Cancer Lett.

[4] Huang EJ, Reichardt LF (2003). Trk receptors: roles in neuronal signal transduction. Annu Rev Biochem.

[5] Amatu A, Sartore-Bianchi A, Siena S (2016). NTRK gene fusions as novel targets of cancer therapy across multiple tumour types. ESMO Open.

[6] Medves S, Demoulin JB (2012). Tyrosine kinase gene fusions in cancer: translating mechanisms into targeted therapies. J Cell Mol Med.

[7] Wai DH, Knezevich SR, Lucas T, Jansen B, Kay RJ, Sorensen PHB (2000). The ETV6-NTRK3 gene fusion encodes a chimeric protein tyrosine kinase that transforms NIH3T3 cells. Oncogene.

[8] Vaishnavi A, Le AT, Doebele RC (2015). TRKing down an old oncogene in a new era of targeted therapy. Cancer Discov.

[9] Triche TJ, Hicks MJ, Sorensen PH (2015) Diagnostic pathology of pediatric malignancies. In: Pizzo PA, Poplack DG (eds) Principles and practice of pediatric oncology, 7th Edition. Wolters Kluwer Health

[10] Martin-Zanca D, Hughes SH, Barbacid M (1986). A human oncogene formed by the fusion of truncated tropomyosin and protein tyrosine kinase sequences. Nature.

[11] Knezevich SR, McFadden DE, Tao W, Lim JF, Sorensen PH (1998). A novel ETV6-NTRK3 gene fusion in congenital fibrosarcoma. Nat Genet.

[12] Knezevich SR, Garnett MJ, Pysher TJ, Beckwith JB, Grundy PE, Sorensen PH (1998). ETV6-NTRK3 gene fusions and trisomy 11 establish a histogenetic link between mesoblastic nephroma and congenital fibrosarcoma. Cancer Res.

[13] Rubin BP, Chen CJ, Morgan TW, Xiao S, Grier HE, Kozakewich HP, Perez-Atayde AR, Fletcher JA (1998). Congenital mesoblastic nephroma t(12;15) is associated with ETV6-NTRK3 gene fusion: cytogenetic and molecular relationship to congenital (infantile) fibrosarcoma. Am J Pathol.

[14] Tognon C, Knezevich SR, Huntsman D, Roskelley CD, Melnyk N, Mathers JA, Becker L, Carneiro F, MacPherson N, Horsman D, Poremba C, Sorensen PH (2002). Expression of the ETV6-NTRK3 gene fusion as a primary event in human secretory breast carcinoma. Cancer Cell.

[15] Eguchi M, Eguchi-Ishimae M, Tojo A, Morishita K, Suzuki K, Sato Y, Kudoh S, Tanaka K, Setoyama M, Nagamura F, Asano S, Kamada N (1999). Fusion of ETV6 to neurotrophin-3 receptor TRKC in acute myeloid leukemia with t(12;15)(p13;q25). Blood.

[16] Leeman-Neill RJ, Kelly LM, Liu P, Brenner AV, Little MP, Bogdanova TI, Evdokimova VN, Hatch M, Zurnadzy LY, Nikiforova MN, Yue NJ, Zhang M, Mabuchi K, Tronko MD, Nikiforov YE (2014). ETV6-NTRK3 is a common chromosomal rearrangement in radiation-associated thyroid cancer. Cancer.

[17] Wu G, Diaz AK, Paugh BS, Rankin SL, Ju B, Li Y, Zhu X, Qu C, Chen X, Zhang J, Easton J, Edmonson M, Ma X, Lu C, Nagahawatte P, Hedlund E, Rusch M, Pounds S, Lin T, Onar-Thomas A, Huether R, Kriwacki R, Parker M, Gupta P, Becksfort J, Wei L, Mulder HL, Boggs K, Vadodaria B, Yergeau D, Russell JC, Ochoa K, Fulton RS, Fulton LL, Jones C, Boop FA, Broniscer A, Wetmore C, Gajjar A, Ding L, Mardis ER, Wilson RK, Taylor MR, Downing JR, Ellison DW, Zhang J, Baker SJ (2014). The genomic landscape of diffuse intrinsic pontine glioma and pediatric non-brainstem high-grade glioma. Nat Genet.

[18] El Demellawy D, Cundiff CA, Nasr A, Ozolek JA, Elawabdeh N, Caltharp SA, Masoudian P, Sullivan KJ, de Nanassy J, Shehata BM (2016). Congenital mesoblastic nephroma: a study of 19 cases using immunohistochemistry and ETV6-NTRK3 fusion gene rearrangement. Pathology.

[19] Skalova A, Vanecek T, Sima R, Laco J, Weinreb I, Perez-Ordonez B, Starek I, Geierova M, Simpson RH, Passador-Santos F, Ryska A, Leivo I, Kinkor Z, Michal M (2010). Mammary analogue secretory carcinoma of salivary glands, containing the ETV6-NTRK3 fusion gene: a hitherto undescribed salivary gland tumor entity. Am J Surg Pathol.

[20] Bourgeois JM, Knezevich SR, Mathers JA, Sorensen PH (2000). Molecular detection of the ETV6-NTRK3 gene fusion differentiates congenital fibrosarcoma from other childhood spindle cell tumors. Am J Surg Pathol.

[21] Sartore-Bianchi A, Ardini E, Bosotti R, Amatu A, Valtorta E, Somaschini A, Raddrizzani L, Palmeri L, Banfi P, Bonazzina E, Misale S, Marrapese G, Leone A, Alzani R, Luo D, Hornby Z, Lim J, Veronese S, Vanzulli A, Bardelli A, Martignoni M, Davite C, Galvani A, Isacchi A, Siena S (2016) Sensitivity to entrectinib associated with a novel LMNA-NTRK1 gene fusion in metastatic colorectal cancer. J Natl Cancer Inst 108(1). 10.1093/jnci/djv306 10.1093/jnci/djv306 PMC4712682 26563355

[22] Drilon A, Siena S, Ou SI, Patel M, Ahn MJ, Lee J, Bauer TM, Farago AF, Wheler JJ, Liu SV, Doebele R, Giannetta L, Cerea G, Marrapese G, Schirru M, Amatu A, Bencardino K, Palmeri L, Sartore-Bianchi A, Vanzulli A, Cresta S, Damian S, Duca M, Ardini E, Li G, Christiansen J, Kowalski K, Johnson AD, Patel R, Luo D, Chow-Maneval E, Hornby Z, Multani PS, Shaw AT, De Braud FG (2017). Safety and antitumor activity of the multitargeted pan-TRK, ROS1, and ALK inhibitor entrectinib: combined results from two phase I trials (ALKA-372-001 and STARTRK-1). Cancer Discov.

[23] Drilon A, Laetsch TW, Kummar S, DuBois SG, Lassen UN, Demetri GD, Nathenson M, Doebele RC, Farago AF, Pappo AS, Turpin B, Dowlati A, Brose MS, Mascarenhas L, Federman N, Berlin J, El-Deiry WS, Baik C, Deeken J, Boni V, Nagasubramanian R, Taylor M, Rudzinski ER, Meric-Bernstam F, Sohal DPS, Ma PC, Raez LE, Hechtman JF, Benayed R, Ladanyi M, Tuch BB, Ebata K, Cruickshank S, Ku NC, Cox MC, Hawkins DS, Hong DS, Hyman DM (2018). Efficacy of larotrectinib in TRK fusion-positive cancers in adults and children. N Engl J Med.

[24] Kummar S, Lassen UN (2018). TRK inhibition: a new tumor-agnostic treatment strategy. Target Oncol.

[25] Lezcano C, Shoushtari AN, Ariyan C, Hollmann TJ, Busam KJ (2018). Primary and metastatic melanoma with NTRK fusions. Am J Surg Pathol.

[26] Prabhakaran N, Guzman MA, Navalkele P, Chow-Maneval E, Batanian JR (2018). Novel TLE4-NTRK2 fusion in a ganglioglioma identified by array-CGH and confirmed by NGS: potential for a gene targeted therapy. Neuropathology.

[27] Chmielecki J, Bailey M, He J, Elvin J, Vergilio JA, Ramkissoon S, Suh J, Frampton GM, Sun JX, Morley S, Spritz D, Ali S, Gay L, Erlich RL, Ross JS, Buxhaku J, Davies H, Faso V, Germain A, Glanville B, Miller VA, Stephens PJ, Janeway KA, Maris JM, Meshinchi S, Pugh TJ, Shern JF, Lipson D (2017). Genomic profiling of a large set of diverse pediatric cancers identifies known and novel mutations across tumor spectra. Cancer Res.

[28] Hechtman JF, Benayed R, Hyman DM, Drilon A, Zehir A, Frosina D, Arcila ME, Dogan S, Klimstra DS, Ladanyi M, Jungbluth AA (2017). Pan-Trk immunohistochemistry is an efficient and reliable screen for the detection of NTRK fusions. Am J Surg Pathol.

[29] Milione M, Ardini E, Christiansen J, Valtorta E, Veronese S, Bosotti R, Pellegrinelli A, Testi A, Pietrantonio F, Fuca G, Wei G, Murphy D, Siena S, Isacchi A, De Braud F (2017). Identification and characterization of a novel SCYL3-NTRK1 rearrangement in a colorectal cancer patient. Oncotarget.

[30] Edgren H OK, Ruusulehto A, Ganji G (2015) Rapid pan-cancer identification of previously unidentified fusion genes to enable novel targeted therapeutics. Proceedings: AACR 106th annual meeting 2015. 75:suppl 15; abstract 4793

[31] Kim J, Lee Y, Cho HJ, Lee YE, An J, Cho GH, Ko YH, Joo KM, Nam DH (2014). NTRK1 fusion in glioblastoma multiforme. PLoS One.

[32] Sigal D, Tartar M, Xavier M, Bao F, Foley P, Luo D, Christiansen J, Hornby Z, Maneval EC, Multani P (2017). Activity of Entrectinib in a patient with the first reported NTRK fusion in neuroendocrine Cancer. Journal of the National Comprehensive Cancer Network : JNCCN.

[33] Stransky N, Cerami E, Schalm S, Kim JL, Lengauer C (2014). The landscape of kinase fusions in cancer. Nat Commun.

[34] Church AJ, Calicchio ML, Nardi V, Skalova A, Pinto A, Dillon DA, Gomez-Fernandez CR, Manoj N, Haimes JD, Stahl JA, Dela Cruz FS, Tannenbaum-Dvir S, Glade-Bender JL, Kung AL, DuBois SG, Kozakewich HP, Janeway KA, Perez-Atayde AR, Harris MH (2018). Recurrent EML4-NTRK3 fusions in infantile fibrosarcoma and congenital mesoblastic nephroma suggest a revised testing strategy. Modern pathology : an official journal of the United States and Canadian academy of. Pathology, Inc.

[35] Lee SJ, Li GG, Kim ST, Hong ME, Jang J, Yoon N, Ahn SM, Murphy D, Christiansen J, Wei G, Hornby Z, Lee DW, Park JO, Park YS, Lim HY, Hong SN, Kim SH, Kang WK, Park K, Park WY, Kim KM, Lee J (2015). NTRK1 rearrangement in colorectal cancer patients: evidence for actionable target using patient-derived tumor cell line. Oncotarget.

[36] Brenca M, Rossi S, Polano M, Gasparotto D, Zanatta L, Racanelli D, Valori L, Lamon S, Dei Tos AP, Maestro R (2016). Transcriptome sequencing identifies ETV6-NTRK3 as a gene fusion involved in GIST. J Pathol.

[37] Yoshihara K, Wang Q, Torres-Garcia W, Zheng S, Vegesna R, Kim H, Verhaak RG (2015). The landscape and therapeutic relevance of cancer-associated transcript fusions. Oncogene.

[38] Jones DT, Hutter B, Jager N, Korshunov A, Kool M, Warnatz HJ, Zichner T, Lambert SR, Ryzhova M, Quang DA, Fontebasso AM, Stutz AM, Hutter S, Zuckermann M, Sturm D, Gronych J, Lasitschka B, Schmidt S, Seker-Cin H, Witt H, Sultan M, Ralser M, Northcott PA, Hovestadt V, Bender S, Pfaff E, Stark S, Faury D, Schwartzentruber J, Majewski J, Weber UD, Zapatka M, Raeder B, Schlesner M, Worth CL, Bartholomae CC, von Kalle C, Imbusch CD, Radomski S, Lawerenz C, van Sluis P, Koster J, Volckmann R, Versteeg R, Lehrach H, Monoranu C, Winkler B, Unterberg A, Herold-Mende C, Milde T, Kulozik AE, Ebinger M, Schuhmann MU, Cho YJ, Pomeroy SL, von Deimling A, Witt O, Taylor MD, Wolf S, Karajannis MA, Eberhart CG, Scheurlen W, Hasselblatt M, Ligon KL, Kieran MW, Korbel JO, Yaspo ML, Brors B, Felsberg J, Reifenberger G, Collins VP, Jabado N, Eils R, Lichter P, Pfister SM, International Cancer Genome Consortium PedBrain Tumor P (2013). Recurrent somatic alterations of FGFR1 and NTRK2 in pilocytic astrocytoma. Nat Genet.

[39] Roberts KG, Li Y, Payne-Turner D, Harvey RC, Yang YL, Pei D, McCastlain K, Ding L, Lu C, Song G, Ma J, Becksfort J, Rusch M, Chen SC, Easton J, Cheng J, Boggs K, Santiago-Morales N, Iacobucci I, Fulton RS, Wen J, Valentine M, Cheng C, Paugh SW, Devidas M, Chen IM, Reshmi S, Smith A, Hedlund E, Gupta P, Nagahawatte P, Wu G, Chen X, Yergeau D, Vadodaria B, Mulder H, Winick NJ, Larsen EC, Carroll WL, Heerema NA, Carroll AJ, Grayson G, Tasian SK, Moore AS, Keller F, Frei-Jones M, Whitlock JA, Raetz EA, White DL, Hughes TP, Guidry Auvil JM, Smith MA, Marcucci G, Bloomfield CD, Mrozek K, Kohlschmidt J, Stock W, Kornblau SM, Konopleva M, Paietta E, Pui CH, Jeha S, Relling MV, Evans WE, Gerhard DS, Gastier-Foster JM, Mardis E, Wilson RK, Loh ML, Downing JR, Hunger SP, Willman CL, Zhang J, Mullighan CG (2014). Targetable kinase-activating lesions in Ph-like acute lymphoblastic leukemia. N Engl J Med.

[40] George J, Walter V, Peifer M, Alexandrov LB, Seidel D, Leenders F, Maas L, Muller C, Dahmen I, Delhomme TM, Ardin M, Leblay N, Byrnes G, Sun R, De Reynies A, McLeer-Florin A, Bosco G, Malchers F, Menon R, Altmuller J, Becker C, Nurnberg P, Achter V, Lang U, Schneider PM, Bogus M, Soloway MG, Wilkerson MD, Cun Y, McKay JD, Moro-Sibilot D, Brambilla CG, Lantuejoul S, Lemaitre N, Soltermann A, Weder W, Tischler V, Brustugun OT, Lund-Iversen M, Helland A, Solberg S, Ansen S, Wright G, Solomon B, Roz L, Pastorino U, Petersen I, Clement JH, Sanger J, Wolf J, Vingron M, Zander T, Perner S, Travis WD, Haas SA, Olivier M, Foll M, Buttner R, Hayes DN, Brambilla E, Fernandez-Cuesta L, Thomas RK (2018). Integrative genomic profiling of large-cell neuroendocrine carcinomas reveals distinct subtypes of high-grade neuroendocrine lung tumors. Nat Commun.

[41] Ronsley R, Rassekh SR, Shen Y, Lee AF, Jantzen C, Halparin J, Albert C, Hawkins DS, Amed S, Rothstein R, Mungall AJ, Dix D, Blair G, Nadel H, Jones SJM, Laskin J, Marra MA, JD R (2018). Application of genomics to identify therapeutic targets in recurrent pediatric papillary thyroid carcinoma. Cold Spring Harb Mol Case Stud.

[42] Hartmaier RJ, Albacker LA, Chmielecki J, Bailey M, He J, Goldberg ME, Ramkissoon S, Suh J, Elvin JA, Chiacchia S, Frampton GM, Ross JS, Miller V, Stephens PJ, Lipson D (2017). High-throughput genomic profiling of adult solid tumors reveals novel insights into Cancer pathogenesis. Cancer Res.

[43] Ross JS, Wang K, Gay L, Al-Rohil R, Rand JV, Jones DM, Lee HJ, Sheehan CE, Otto GA, Palmer G, Yelensky R, Lipson D, Morosini D, Hawryluk M, Catenacci DV, Miller VA, Churi C, Ali S, Stephens PJ (2014). New routes to targeted therapy of intrahepatic cholangiocarcinomas revealed by next-generation sequencing. Oncologist.

[44] Shi E, Chmielecki J, Tang CM, Wang K, Heinrich MC, Kang G, Corless CL, Hong D, Fero KE, Murphy JD, Fanta PT, Ali SM, De Siena M, Burgoyne AM, Movva S, Madlensky L, Heestand GM, Trent JC, Kurzrock R, Morosini D, Ross JS, Harismendy O, Sicklick JK (2016). FGFR1 and NTRK3 actionable alterations in "wild-type" gastrointestinal stromal tumors. J Transl Med.

[45] Wiesner T, He J, Yelensky R, Esteve-Puig R, Botton T, Yeh I, Lipson D, Otto G, Brennan K, Murali R, Garrido M, Miller VA, Ross JS, Berger MF, Sparatta A, Palmedo G, Cerroni L, Busam KJ, Kutzner H, Cronin MT, Stephens PJ, Bastian BC (2014). Kinase fusions are frequent in Spitz tumours and spitzoid melanomas. Nat Commun.

[46] Zheng Z, Liebers M, Zhelyazkova B, Cao Y, Panditi D, Lynch KD, Chen J, Robinson HE, Shim HS, Chmielecki J, Pao W, Engelman JA, Iafrate AJ, Le LP (2014). Anchored multiplex PCR for targeted next-generation sequencing. Nat Med.

[47] Liang J, Cai W, Feng D, Teng H, Mao F, Jiang Y, Hu S, Li X, Zhang Y, Liu B, Sun ZS (2018). Genetic landscape of papillary thyroid carcinoma in the Chinese population. J Pathol.

[48] Vaishnavi A, Capelletti M, Le AT, Kako S, Butaney M, Ercan D, Mahale S, Davies KD, Aisner DL, Pilling AB, Berge EM, Kim J, Sasaki H, Park S, Kryukov G, Garraway LA, Hammerman PS, Haas J, Andrews SW, Lipson D, Stephens PJ, Miller VA, Varella-Garcia M, Janne PA, Doebele RC (2013). Oncogenic and drug-sensitive NTRK1 rearrangements in lung cancer. Nat Med.

[49] Chiang S, Cotzia P, Hyman DM, Drilon A, Tap WD, Zhang L, Hechtman JF, Frosina D, Jungbluth AA, Murali R, Park KJ, Soslow RA, Oliva E, Iafrate AJ, Benayed R, Ladanyi M, Antonescu CR (2018). NTRK fusions define a novel uterine sarcoma subtype with features of Fibrosarcoma. Am J Surg Pathol.

[50] Tognon C, Garnett M, Kenward E, Kay R, Morrison K, Sorensen PHB (2001). The chimeric protein tyrosine kinase ETV6-NTRK3 requires both Ras-Erk1/2 and PI3-kinase-Akt signaling for fibroblast transformation. Cancer Res.

[51] Tognon CE, Mackereth CD, Somasiri AM, McIntosh LP, Sorensen PHB (2004). Mutations in the SAM domain of the ETV6-NTRK3 chimeric tyrosine kinase block polymerization and transformation activity. Mol Cell Biol.

[52] Li Z, Tognon CE, Godinho FJ, Yasaitis L, Hock H, Herschkowitz JI, Lannon CL, Cho E, Kim SJ, Bronson RT, Perou CM, Sorensen PH, Orkin SH (2007). ETV6-NTRK3 fusion oncogene initiates breast cancer from committed mammary progenitors via activation of AP1 complex. Cancer Cell.

[53] Morrison KB, Tognon CE, Garnett MJ, Deal C, Sorensen PH (2002). ETV6-NTRK3 transformation requires insulin-like growth factor 1 receptor signaling and is associated with constitutive IRS-1 tyrosine phosphorylation. Oncogene.

[54] Martin MJ, Melnyk N, Pollard M, Bowden M, Leong H, Podor TJ, Gleave M, Sorensen PH (2006). The insulin-like growth factor I receptor is required for Akt activation and suppression of anoikis in cells transformed by the ETV6-NTRK3 chimeric tyrosine kinase. Mol Cell Biol.

[55] Tognon CE, Rafn B, Cetinbas NM, Kamura T, Trigo G, Rotblat B, Okumura F, Matsumoto M, Chow C, Davare M, Pollak M, Mayor T, Sorensen PH (2018). Insulin-like growth factor 1 receptor stabilizes the ETV6-NTRK3 chimeric oncoprotein by blocking its KPC1/Rnf123-mediated proteasomal degradation. J Biol Chem.

[56] Kheder ES, Hong DS (2018). Emerging targeted therapy for tumors with NTRK fusion proteins. Clin Cancer Res:Clincanres.

[57] Liu D, Offin M, Harnicar S, Li BT, Drilon A (2018). Entrectinib: an orally available, selective tyrosine kinase inhibitor for the treatment of NTRK, ROS1, and ALK fusion-positive solid tumors. Ther Clin Risk Manag.

[58] Ziegler DS, Wong M, Mayoh C, Kumar A, Tsoli M, Mould E, Tyrrell V, Khuong-Quang DA, Pinese M, Gayevskiy V, Cohn RJ, Lau LMS, Reynolds M, Cox MC, Gifford A, Rodriguez M, Cowley MJ, Ekert PG, Marshall GM, Haber M (2018). Brief report: potent clinical and radiological response to larotrectinib in TRK fusion-driven high-grade glioma. Br J Cancer.

[59] Laetsch TW, DuBois SG, Mascarenhas L, Turpin B, Federman N, Albert CM, Nagasubramanian R, Davis JL, Rudzinski E, Feraco AM, Tuch BB, Ebata KT, Reynolds M, Smith S, Cruickshank S, Cox MC, Pappo AS, Hawkins DS (2018). Larotrectinib for paediatric solid tumours harbouring NTRK gene fusions: phase 1 results from a multicentre, open-label, phase 1/2 study. The Lancet Oncology.

[60] U.S. Food & Drug Administration. Drugs@FDA: FDA Approved Drug Products. https://www.accessdata.fda.gov/scripts/cder/daf/index.cfm?event=overview.process&varApplNo=210861. [Accessed 12 March 2019]

[61] Cui C, Shu W, Li P (2016). Fluorescence in situ hybridization: cell-based genetic diagnostic and research applications. Frontiers in cell and developmental biology.

[62] Argani P, Fritsch M, Kadkol SS, Schuster A, Beckwith JB, Perlman EJ (2000) Detection of the ETV6-NTRK3 chimeric RNA of infantile fibrosarcoma/cellular congenital mesoblastic nephroma in paraffin-embedded tissue: application to challenging pediatric renal stromal tumors. Modern pathology : an official journal of the United States and Canadian academy of Pathology, Inc 13 (1):29–36. 10.1038/modpathol.3880006 10.1038/modpathol.3880006 10658907

[63] Kumar S, Razzaq SK, Vo AD, Gautam M, Li H (2016). Identifying fusion transcripts using next generation sequencing. Wiley interdisciplinary reviews RNA.

[64] Doshi S, Ray D, Stein K, Zhang J, Koduru P, Fogt F, Wellman A, Wat R, Mathews C (2016) Economic analysis of alternative strategies for detection of ALK rearrangements in non small cell lung Cancer. Diagnostics (Basel) 6(1). 10.3390/diagnostics6010004 10.3390/diagnostics6010004 PMC4808819 26838801

[65] Davies KD, Le AT, Sheren J, Nijmeh H, Gowan K, Jones KL, Varella-Garcia M, Aisner DL, Doebele RC (2018). Comparison of molecular testing modalities for detection of ROS1 rearrangements in a cohort of positive patient samples. J Thorac Oncol.

[66] Godbey P, Myles JL, Black-Schaffer WS, Scott J Pathology payment – An overview of the 2018 proposed Medicare physician fee schedule. College of American Pathologists https://documents.cap.org/documents/pathology-payment.pdf. [Accessed 12 March 2019]

[67] Helman E, Nguyen M, Karlovich CA, Despain D, Choquette AK, Spira AI, Yu HA, Camidge DR, Harding TC, Lanman RB, Simmons AD (2018). Cell-free DNA Next-generation sequencing prediction of response and resistance to third-generation EGFR inhibitor. Clin Lung Cancer.

[68] Bubendorf L, Buttner R, Al-Dayel F, Dietel M, Elmberger G, Kerr K, Lopez-Rios F, Marchetti A, Oz B, Pauwels P, Penault-Llorca F, Rossi G, Ryska A, Thunnissen E (2016). Testing for ROS1 in non-small cell lung cancer: a review with recommendations. Virchows Arch.

[69] Cheng DT, Mitchell TN, Zehir A, Shah RH, Benayed R, Syed A, Chandramohan R, Liu ZY, Won HH, Scott SN, Brannon AR, O'Reilly C, Sadowska J, Casanova J, Yannes A, Hechtman JF, Yao J, Song W, Ross DS, Oultache A, Dogan S, Borsu L, Hameed M, Nafa K, Arcila ME, Ladanyi M, Berger MF (2015). Memorial Sloan Kettering-integrated mutation profiling of actionable Cancer targets (MSK-IMPACT). a hybridization capture-based next-generation sequencing clinical assay for solid tumor molecular oncology J Mol Diagn.

[70] Mullin E Next Generation sequencing in the clinical laboratory: in the trenches with early adopters. https://www.aacc.org/publications/cln/articles/2015/may/next-generation-sequencing-in-the-clinical-laboratory. [Accessed 12 March 2019]

[71] Sabari JK, Santini F, Bergagnini I, Lai WV, Arbour KC, Drilon A (2017). Changing the therapeutic landscape in non-small cell lung cancers: the evolution of comprehensive molecular profiling improves access to therapy. Curr Oncol Rep.

[72] Paasinen-Sohns A, Koelzer VH, Frank A, Schafroth J, Gisler A, Sachs M, Graber A, Rothschild SI, Wicki A, Cathomas G, Mertz KD (2017). Single-center experience with a targeted Next generation sequencing assay for assessment of relevant somatic alterations in solid tumors. Neoplasia.

[73] Wegert J, Vokuhl C, Collord G, Del Castillo Velasco-Herrera M, Farndon SJ, Guzzo C, Jorgensen M, Anderson J, Slater O, Duncan C, Bausenwein S, Streitenberger H, Ziegler B, Furtwangler R, Graf N, Stratton MR, Campbell PJ, Jones DT, Koelsche C, Pfister SM, Mifsud W, Sebire N, Sparber-Sauer M, Koscielniak E, Rosenwald A, Gessler M, Behjati S (2018). Recurrent intragenic rearrangements of EGFR and BRAF in soft tissue tumors of infants. Nat Commun.

[74] van den Akker J, Mishne G, Zimmer AD, Zhou AY (2018). A machine learning model to determine the accuracy of variant calls in capture-based next generation sequencing. BMC Genomics.

[75] Tkachuk DC, Westbrook CA, Andreeff M, Donlon TA, Cleary ML, Suryanarayan K, Homge M, Redner A, Gray J, Pinkel D (1990). Detection of bcr-abl fusion in chronic myelogeneous leukemia by in situ hybridization. Science.

[76] Doebele RC, Davis LE, Vaishnavi A, Le AT, Estrada-Bernal A, Keysar S, Jimeno A, Varella-Garcia M, Aisner DL, Li Y, Stephens PJ, Morosini D, Tuch BB, Fernandes M, Nanda N, Low JA (2015). An oncogenic NTRK fusion in a patient with soft-tissue sarcoma with response to the tropomyosin-related kinase inhibitor LOXO-101. Cancer Discov.

[77] Lyu X, Wang X, Zhang L, Chen Z, Zhao Y, Hu J, Fan R, Song Y (2017). Detection of 22 common leukemic fusion genes using a single-step multiplex qRT-PCR-based assay. Diagn Pathol.

[78] Wu YC, Chang IC, Wang CL, Chen TD, Chen YT, Liu HP, Chu Y, Chiu YT, Wu TH, Chou LH, Chen YR, Huang SF (2013). Comparison of IHC, FISH and RT-PCR methods for detection of ALK rearrangements in 312 non-small cell lung cancer patients in Taiwan. PLoS One.

[79] Beadling C, Wald AI, Warrick A, Neff TL, Zhong S, Nikiforov YE, Corless CL, Nikiforova MN (2016). A multiplexed amplicon approach for detecting gene fusions by Next-generation sequencing. J Mol Diagn.

[80] Fehr A, Loning T, Stenman G (2011). Mammary analogue secretory carcinoma of the salivary glands with ETV6-NTRK3 gene fusion. Am J Surg Pathol.

[81] Gatalica Z, Xiu J, Swensen J, Vranic S (2018) Molecular characterization of cancers with NTRK gene fusions. Modern pathology : an official journal of the United States and Canadian Academy of Pathology, Inc 10.1038/s41379-018-0118-3 10.1038/s41379-018-0118-3 30171197

[82] Zhang J, White NM, Schmidt HK, Fulton RS, Tomlinson C, Warren WC, Wilson RK, Maher CA (2016). INTEGRATE: gene fusion discovery using whole genome and transcriptome data. Genome Res.

[83] Maher CA, Kumar-Sinha C, Cao X, Kalyana-Sundaram S, Han B, Jing X, Sam L, Barrette T, Palanisamy N, Chinnaiyan AM (2009). Transcriptome sequencing to detect gene fusions in cancer. Nature.

[84] Farago AF, Le LP, Zheng Z, Muzikansky A, Drilon A, Patel M, Bauer TM, Liu SV, Ou SH, Jackman D, Costa DB, Multani PS, Li GG, Hornby Z, Chow-Maneval E, Luo D, Lim JE, Iafrate AJ, Shaw AT (2015). Durable clinical response to entrectinib in NTRK1-rearranged non-small cell lung cancer. J Thorac Oncol.

[85] Alvarez-Breckenridge C, Miller JJ, Nayyar N, Gill CM, Kaneb A, D'Andrea M, Le LP, Lee J, Cheng J, Zheng Z, Butler WE, Multani P, Chow Maneval E, Ha Paek S, Toyota BD, Dias-Santagata D, Santagata S, Romero J, Shaw AT, Farago AF, Yip S, Cahill DP, Batchelor TT, Iafrate AJ, Brastianos PK (2017). Clinical and radiographic response following targeting of BCAN-NTRK1 fusion in glioneuronal tumor. NPJ Precis Oncol.

[86] Murphy DA, Ely HA, Shoemaker R, Boomer A, Culver BP, Hoskins I, Haimes JD, Walters RD, Fernandez D, Stahl JA, Lee J, Kim KM, Lamoureux J, Christiansen J (2017). Detecting gene rearrangements in patient populations through a 2-step diagnostic test comprised of rapid IHC enrichment followed by sensitive next-generation sequencing. Appl Immunohistochem Mol Morphol.

[87] Qadir MA, Zhan SH, Kwok B, Bruestle J, Drees B, Popescu OE, Sorensen PH (2014). ChildSeq-RNA. a next-generation sequencing-based diagnostic assay to identify known fusion transcripts in childhood sarcomas J Mol Diagn.

[88] Chang KTE, Goytain A, Tucker T, Karsan A, Lee CH, Ng TL, Nielsen TO (2018). Development and evaluation of a pan-sarcoma fusion gene detection assay using the NanoString nCounter platform. J Mol Diagn.

[89] Lindquist KE, Karlsson A, Leveen P, Brunnstrom H, Reutersward C, Holm K, Jonsson M, Annersten K, Rosengren F, Jirstrom K, Kosieradzki J, Ek L, Borg A, Planck M, Jonsson G, Staaf J (2017). Clinical framework for next generation sequencing based analysis of treatment predictive mutations and multiplexed gene fusion detection in non-small cell lung cancer. Oncotarget.

[90] Hyman DM, Solit DB, Arcila ME, Cheng DT, Sabbatini P, Baselga J, Berger MF, Ladanyi M (2015). Precision medicine at memorial Sloan Kettering Cancer center: clinical next-generation sequencing enabling next-generation targeted therapy trials. Drug Discov Today.

